# Methods for preparing polymer-decorated single exchange-biased magnetic nanoparticles for application in flexible polymer-based films

**DOI:** 10.3762/bjnano.8.43

**Published:** 2017-02-09

**Authors:** Laurence Ourry, Delphine Toulemon, Souad Ammar, Fayna Mammeri

**Affiliations:** 1 Université Paris Diderot, Sorbonne Paris Cité, CNRS UMR 7086 ITODYS, Case 7090, 5 rue Thomas Mann, Paris, France

**Keywords:** assembly, ATRP, magnetic nanoparticle, exchange-bias, films, functionalization, polymerization, poly(methyl methacrylate), polystyrene, seed-mediated growth, surface

## Abstract

**Background:** Magnetic nanoparticles (NPs) must not only be well-defined in composition, shape and size to exhibit the desired properties (e.g., exchange-bias for thermal stability of the magnetization) but also judiciously functionalized to ensure their stability in air and their compatibility with a polymer matrix, in order to avoid aggregation which may seriously affect their physical properties. Dipolar interactions between NPs too close to each other favour a collective magnetic glass state with lower magnetization and coercivity because of inhomogeneous and frustrated macrospin cluster freezing. Consequently, tailoring chemically (through surface functionalization) and magnetically stable NPs for technological applications is of primary importance.

**Results:** In this work, well-characterized exchange-biased perfectly epitaxial Co*_x_*Fe_3−_*_x_*O_4_@CoO core@shell NPs, which were isotropic in shape and of about 10 nm in diameter, were decorated by two different polymers, poly(methyl methacrylate) (PMMA) or polystyrene (PS), using radical-controlled polymerization under various processing conditions. We compared the influence of the synthesis parameters on the structural and microstructural properties of the resulting hybrid systems, with special emphasis on significantly reducing their mutual magnetic attraction. For this, we followed two routes: the first one consists of the direct grafting of bromopropionyl ester groups at the surface of the NPs, which were previously recovered and redispersed in a suitable solvent. The second route deals with an “all in solution” process, based on the decoration of NPs by oleic acid followed by ligand exchange with the desired bromopropionyl ester groups. We then built various assemblies of NPs directly on a substrate or suspended in PMMA.

**Conclusion:** The alternative two-step strategy leads to better dispersed polymer-decorated magnetic particles, and the resulting nanohybrids can be considered as valuable building blocks for flexible, magnetic polymer-based devices.

## Introduction

Polymer-based hybrid materials are opening the way for engineering new, multifunctional, flexible materials exhibiting novel properties (e.g., mechanical, magnetic, electrical, optical) due to the synergy between the two components, polymer and inorganic nanoparticles (NPs) [1]. In the case of magnetic hybrids, one of the main challenges is to avoid NP aggregation. The magnetic response of NPs to external magnetic stimulus depends strongly on their intrinsic properties (composition, size, and shape) but also on their spatial arrangement (self-assembly, dispersion in organic media, compatibility with polymers) [2–3]. If the interparticle distances are decreased too much, a collective magnetic glass state is set up, reducing magnetization and coercivity because of inhomogeneous and frustrated macrospin cluster freezing [4–6]. Ideally, flexible magnetic devices require dense but well-separated magnetic NPs to decrease interparticle interactions, particularly dipolar ones [7–8].

The general strategy for such a purpose consists of forming core–shell hybrid structures in which the shell consists of a corona of polymer chains grafted onto the inorganic NP surface. Among the available polymer grafting processes, living-radical polymerization (e.g., atom-transfer radical polymerization (ATRP), reversible addition–fragmentation chain transfer (RAFT) or nitroxide-mediated polymerization (NMP)) makes it possible to establish robust polymer–particle bonds and then grow polymer brushes of controlled molecular weight and polydispersity with a satisfactory grafting density. The polymer chains also stabilize the inorganic NPs with respect to the ambient atmosphere and provide compatibility with the resulting polymer matrix.

This strategy has been widely investigated with magnetic NPs. To date, most NPs studied were of iron oxide [9–14]. Exchange-biased NPs (ENPs) have been scarcely considered [15] despite their improved magnetic properties. These particles consist of ferro- or ferrimagnetic (F) cores coated with nanocrystalline antiferromagnetic (AF) layers, and exhibit exchange coupling at the F–AF interface (see for instance [16–18]), leading to an enhanced effective magnetic anisotropy constant (*K*_eff_) and a higher temperature of transition from a magnetically blocked state to a superparamagnetic one (*T*_B_) [19–20]. Focusing on such particles, in this work, we propose various material processing routes to prepare weakly interacting and densely arranged hybrid ENPs. The ENPs used were prepared by seed-mediated growth in a polyol medium; they consist of ferrimagnetic Co*_x_*Fe_3−_*_x_*O_4_ single crystals, almost isotropic in shape and of about 10 nm in diameter, coated in a perfectly epitaxial fashion with an antiferromagnetic CoO polycrystalline shell about 1 nm thick, as described in previous work [16]. We then controlled their surface functionality to tentatively design well-tailored nano-building-blocks for the aforementioned devices. To reduce mutual magnetic attraction and aggregation as far as possible in the first stage of polymer grafting, mechanical stirring and dilute suspensions of reactants were used, even if the functionalization of large amounts of particles becomes difficult. We specifically graft poly(methyl methacrylate) (PMMA) and polystyrene (PS) chains around Co*_x_*Fe_3−_*_x_*O_4_@CoO. Several key parameters have to be taken into account to realize a controlled polymerization reaction and especially when one aims to graft an ATRP initiator at the surface of particles: the nature of the surface (e.g., oxide or metal) and the interface between the components of the resulting hybrid, namely polymer chains and inorganic NPs (e.g., covalent, ionic, van der Waals). Several halogenated coupling agents can be used to covalently graft an organic group onto the surface of oxide NPs, e.g., organosilanes [21–23], carboxylate [24–25] or phosphonate/phosphate molecules [26–27].

Organosilanes present the disadvantage of condensing after hydrolysis and leading to a thin shell of polysiloxane whose structure and thickness cannot be well controlled. Carboxylates can be degrafted in solution [28], and are, consequently, not the best candidates for the desired applications. Phosphates and phosphonates are suitable for functionalizing iron and silicon oxide surfaces [29–30] through covalent bonds, in mono-, bi- or tridentate modes; but to date, they have not been used for cobalt oxide and ferrite surfaces.

**Figure 1:**
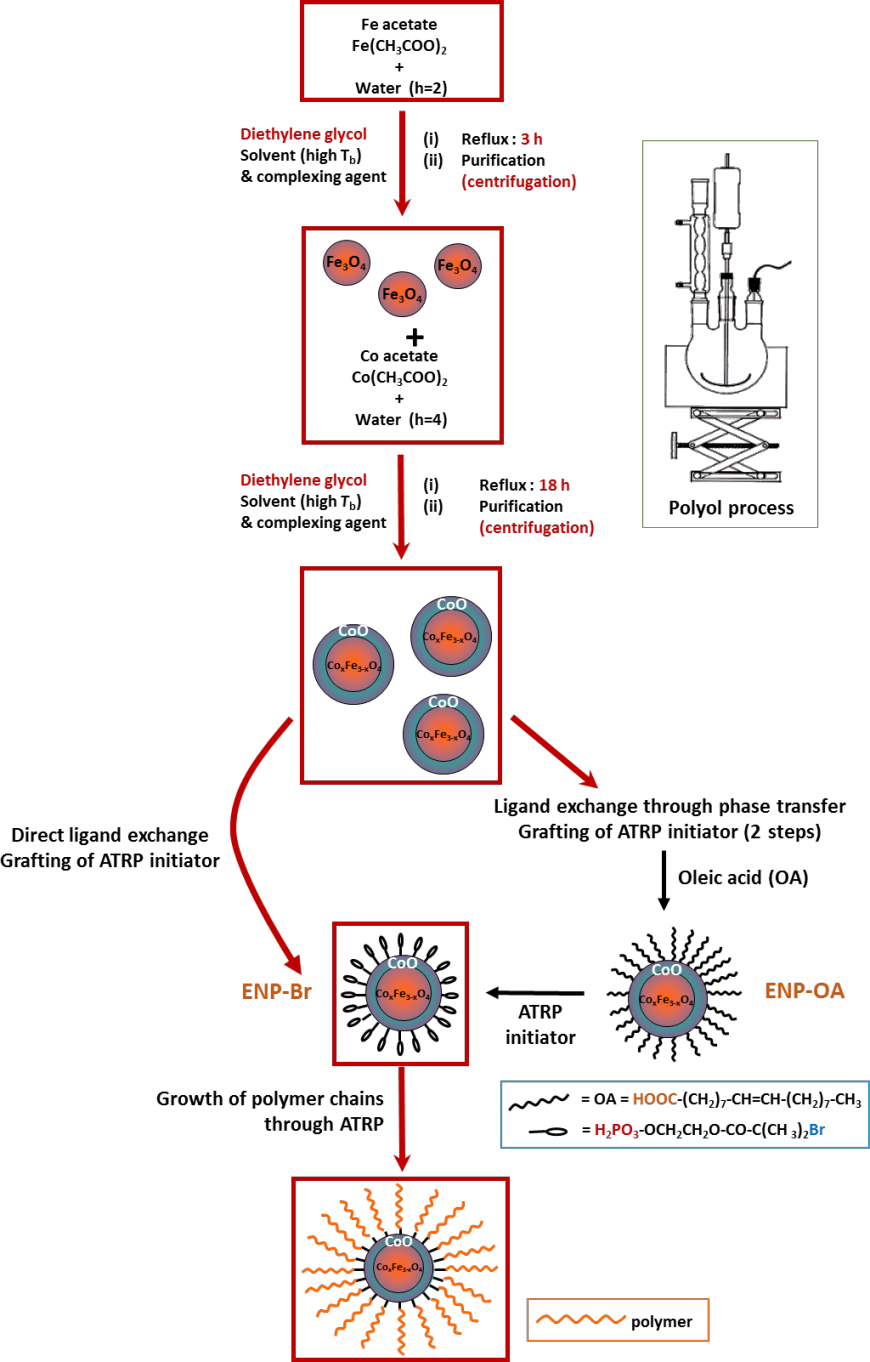
Polymer-decorated exchange-biased Co*_x_*Fe_3−_*_x_*O_4_@CoO magnetic nanoparticles synthesis.

In the case of PMMA, we followed two routes: the first one consists of a direct grafting of bromopropionyl ester molecules ob the surface of ENPs that were previously recovered and redispersed in a suitable solvent. The second uses an “all in solution” process, based on a prior decoration of ENPs by oleic acid ligands, which were subsequently exchanged by the desired bromopropionyl ester species. In the case of PS, we followed exclusively the first route, but in all the cases, the polymer chains were grown by ATRP (see the general synthesis scheme summarized in Figure 1), acting on the NP/monomer weight ratio and on the polymerization time parameters. The resulting nanohybrids were then characterized with special emphasis on the effect of the reaction parameters on their main microstructural properties and taking into account the fact that PS polymerizes more slowly than PMMA.

## Results and Discussion

We chose to work on two very common thermoplastic polymers: PMMA and PS, which have very similar properties (e.g., specific temperatures, mechanical properties, density) but rather different side chains; PMMA has an aliphatic ester group while PS has a more rigid aromatic ring. However, despite their structural and reactivity differences, we followed the same reaction pathway to elaborate ENPs decorated by the two polymers (Figure 1).

### Growth of PMMA chains

We previously developed a two-step pathway to produce the hybrid polymer-decorated ENPs [15]. First, an ATRP initiator, 2-phosphonooxy-2-bromo-2-methylpropanoate, was grafted onto the ENP surface. Under the conditions used (see Experimental), the grafting density of the initiator molecules is 2.8 molecules nm^−2^, in good agreement with previous studies [27]. Then, the PMMA chains were grown by ATRP at 30 °C in the presence of cuprous bromide and *N*,*N*,*N*′,*N*′,*N*′′-pentamethyldiethylenetriamine (PMDTA) to form the catalytic Cu-PMDETA complex, with polymerization times of 1 to 3 h. The polymer coating was characterized by X-ray photoelectron spectroscopy (XPS) and thermogravimetric analysis (TGA) [15]. For all samples the grafted chain density was 1.4 chain nm^−2^, i.e., significantly higher than the results previously reported [15]. A chain density ranging between 0.1 and 1 chain nm^−2^ is commonly reported [13,26,31–32]. In the present case, a higher grafting density means better protection of the magnetic particles against oxidation and more stable magnetic properties over time [33]. Indeed, the aim here is not necessarily to grow very long polymer chains which are diamagnetic, but to functionalize ENPs efficiently in order to increase their compatibility with polymer matrices. Comparison of the grafting densities obtained here with those reported elsewhere suggests that, for ENPs of similar size, phosphates and phosphonates are better grafted and in greater quantity to the surface of ENPs than carboxylates and organosilanes.

### Growth of PS chains

Styrene polymerizes more slowly than methyl methacrylate. Yousi et al. [34] demonstrated that the propagation rate, for similar conversion yields, can be increased by catalysts. Masson et al. [35] reported an increase in the styrene polymerization rate, using malonitrile as a catalyst and from initiator molecules anchored on iron oxide NPs. However, it appears that bonding the initiator to the surface through a phosphonate group limits the rate, reducing the effect of the catalyst. The length of the carbon backbone of the initiator (between phosphate and α-bromo-ester functions) is very important to the ATRP polymerization rate when initiators are directly anchored to the particle surface. Sunday et al. [36] reported that long alkyl chains (16 carbon atoms) or short ones (3 carbon atoms) lead to higher polymerization rates and grafting densities than intermediate chains (e.g., 11 carbon atoms). Masson et al. used an 11-carbon-long initiator. That which we used, the same as for PMMA (2-phosphonooxy-2-bromo-2-methylpropanoate), has only two carbon atoms, and might be expected to give good polymerization rates.

**Figure 2:**
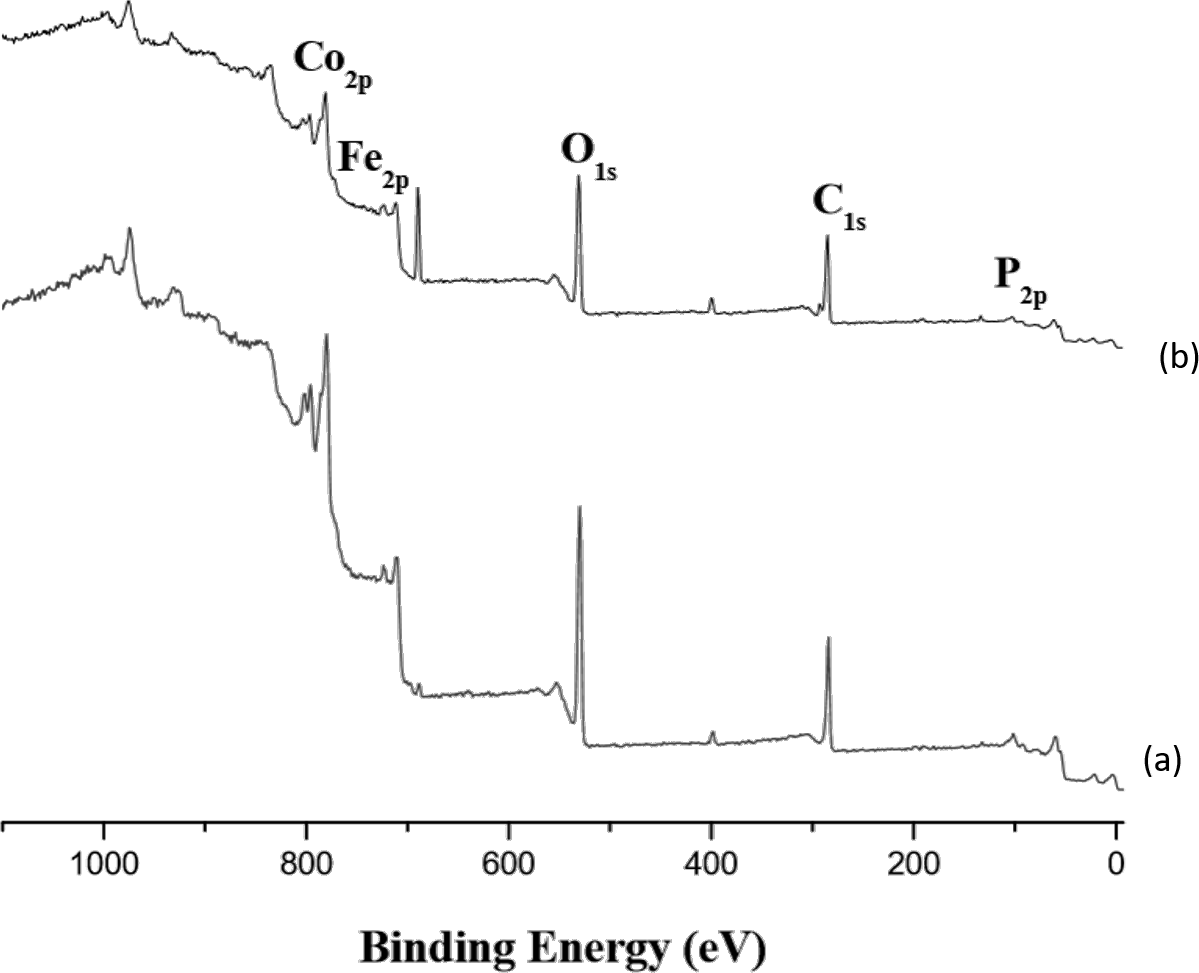
XPS survey spectra of (a) ENP-PS-18, (b) ENP-PS-24.

Working under the same conditions previously used for PMMA growth on ENPs, we prepared a series of samples by varying the polymerization time from 18 to 24 h; the resulting hybrids will be referred to as ENP-PS-h (where h corresponds to the polymerization time in hours). The survey XPS spectra of the resulting hybrids evidence all the characteristic signals of ENPs and initiator species (Figure 2). Hence, the O 1s (531.0 eV), Fe 2p_3/2_ (711.5 eV), Co 2p_3/2_ (781.7 eV), P 2p (133.5eV), and Br 3p_3/2_ (190.7 eV) peaks can be assigned to Co*_x_*Fe_3−_*_x_*O_4_@CoO and PO(OH)_2_O(CH_2_)_2_OCOC(CH_3_)_2_Br phases, respectively. The absence of Cu signals in the ENP-PS spectra suggests that all metallic Cu complexes were eliminated during the washing and purification steps. Chemical compositions (provided by XPS data) of as-produced NPs and ENP-PS are listed in Table 1.

**Table 1:** XPS-determined atomic composition of as-prepared Co*_x_*Fe_3−_*_x_*O_4_-CoO nanoparticles (ENP) and PS-based hybrids.

Sample	Elemental atomic composition (%)					
	Fe	Co	O	C	P	Br

ENP (Co*_x_*Fe_3−_*_x_*O_4_@CoO)*	14.5	24.3	40.2	21.0	–	–
ENP-PS-18	7.7	10.5	37.6	41.5	2.3	0.4
ENP-PS-24	7.4	11.6	37.7	41.8	1.2	0.3

*Reproduced in part with permission from [15]. Copyright 2016 The Royal Society of Chemistry.

**Figure 3:**
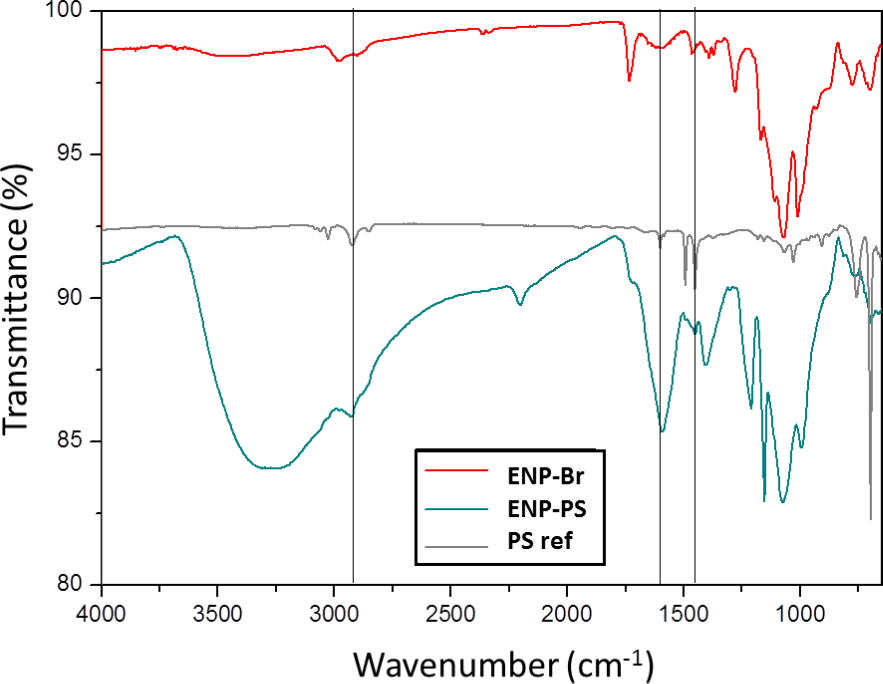
IR spectrum of ENP-PS-18 and those of free PS and ENPs functionalized by the ATRP initiator only (ENP-Br).

The C 1s peak prior to functionalization indicates that organic residues (polyol and acetate molecules) are present at the core–shell NP surface. The Co 2p and Fe 2p peaks are much weaker after polymerization due the presence of a significant polymer coating. The decomposition of the C 1s peak for polystyrene (PS) was difficult since there are many sources of organic matter (PS, initiator, residual polyol and acetates); however, unfortunately, styrene contains no other element suitable for XPS analysis. However, IR spectra of PS-decorated ENPs (Figure 3) exhibit several peaks characteristic of polystyrene: 3024 cm^−1^ (ν_as(CH2_arom)_), 2950 cm^−1^ (ν_s(CH___aliph)_) and 1600 cm^−1^ (ν_C=C_) when compared to a commercial reference.

**Figure 4:**
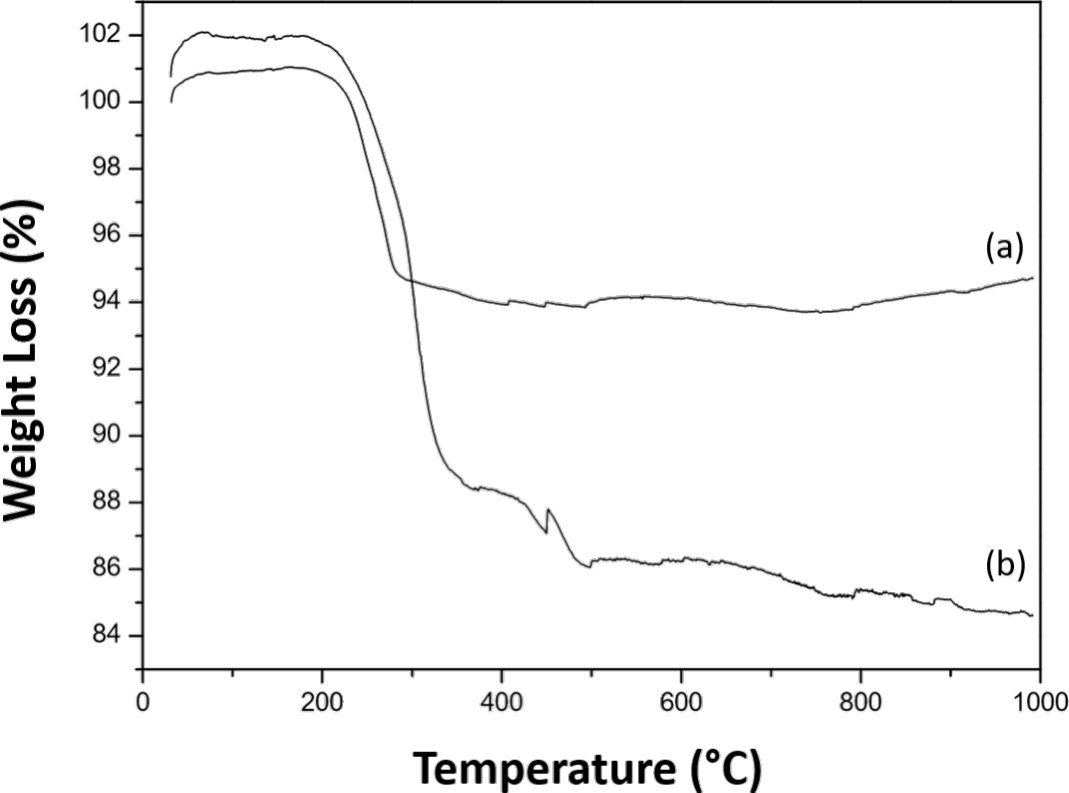
TGA curves of (a) ENP-PS-18 and (b) ENP-PS-20.

Similar to the PMMA-based nanohybrids, the amount of cobalt was found to be higher than that of iron for PS-functionalized ENPs. The mean free paths of 1.5 nm for iron in CoO and 1.4 nm for cobalt in CoO were calculated following the method described previously [15]. However, electrons pass only through 0.5 nm of the Fe-rich core after having penetrated the 1 nm thick CoO shell, leading to a difference in depth analysis of the core and the shell. Then, we assumed that cobalt and carbon are representative of the CoO shell [30] and the PS brushes, respectively, to determine the PS thickness from the C 1s and Co 2p peaks and their relative intensities. The thicknesses were found to be 2.5 ± 0.5 nm for ENP-PS-18 and 3.0 ± 0.5 nm for ENP-PS-24. However, in the XPS chamber (under ultravacuum conditions), the polymer chains tend to collapse. Therefore, one must consider that XPS measurements do not allow the exact chain length to be determined but that of a polymer in a random coil conformation. Nevertheless, we can conclude that more polystyrene is present around the ENP when the polymerization time increases. TGA thermograms of PS-decorated ENPs are presented in Figure 4. PS is generally decomposed at about 380 °C. It can be seen that, although the polymer is thicker at the ENP surface in the case of PS, the weight losses of PS (6% and 14% for NP-PS-18 and NP-PS-24, respectively) are lower than those of PMMA in ENP-PMMA-1 and ENP-PMMA-3 [15], meaning that PS collapses less than PMMA. This is doubtful due to the presence of aromatic rings.

**Figure 5:**
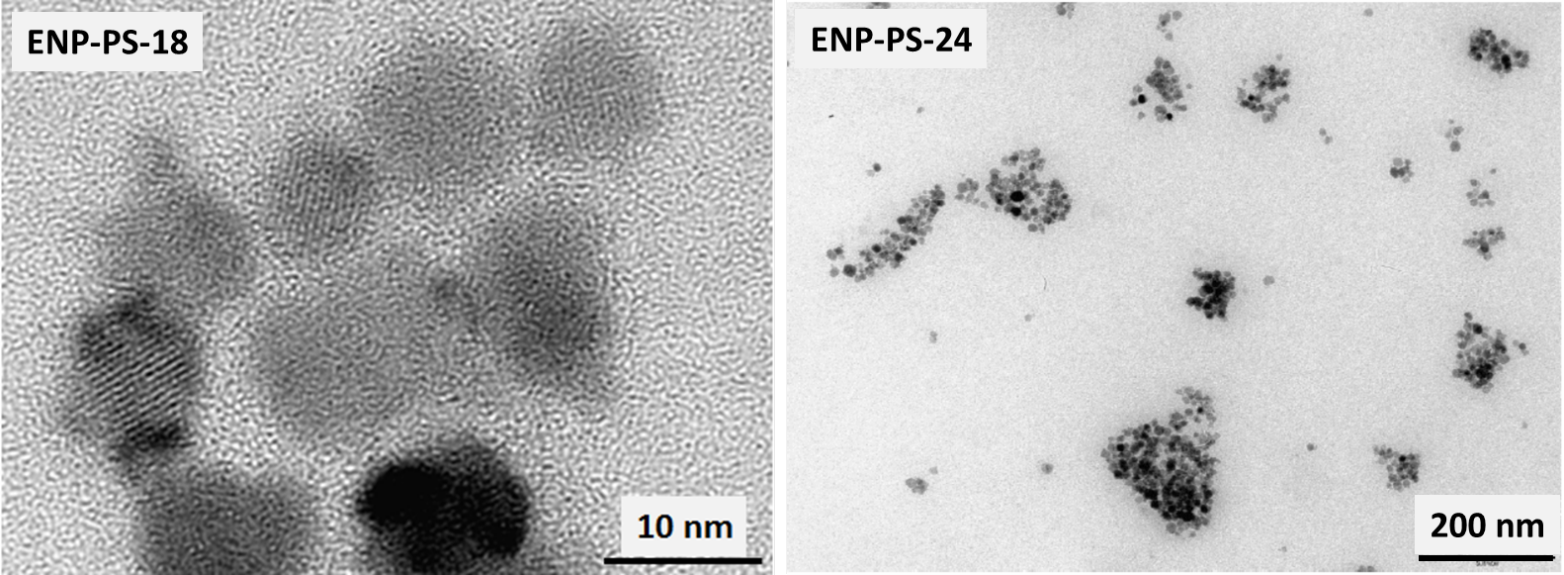
TEM images of ENP-PS-18 (left) and ENP-PS-24 (right).

TEM images presented in Figure 5 depict well-dispersed nanoparticles, although the surface of the grid was not totally covered with ENPs. Similar images have been obtained for the two samples. There are spaces between the ENPs that are close to each other, suggesting that they are separated by PS coatings.

### Ligand exchange for improved separation of magnetic NPs

**Figure 6:**
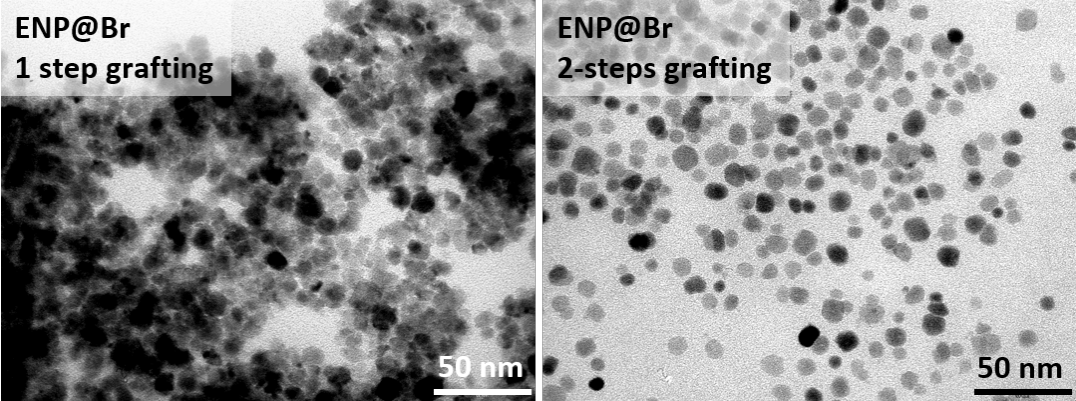
TEM images of a) ENP-Br obtained after centrifugation of as-prepared ENPs and direct grafting of ATRP initiator; b) ENP-Br obtained by two-step ligand exchange by oleic acid followed by grafting of ATRP initiator.

In the previous sections, we demonstrated the feasibility of preparing polymer (PMMA or PS) functionalized magnetic particles with improved polymer chain grafting densities than those previously reported. However, it is still a serious challenge to separate all the particles and avoid a few aggregates. Hence, we replaced the centrifugation of the ENPs after the polyol synthesis by direct ligand exchange between adsorbed polyol molecules and oleic acid (OA) in the reaction mixture (see Experimental). Oleic acid is known to cap oxide nanoparticles by ionic bonding [24–25]. Moreover, it bears a double bond C=C, inducing a degree of structural rigidity and a kink in the chain, promoting the spacing of the particles during their organization. TEM images (Figure 6) clearly show a better separation of the ENPs previously coated with OA instead of being recovered simply by centrifugation of the polyol mixture.

**Figure 7:**
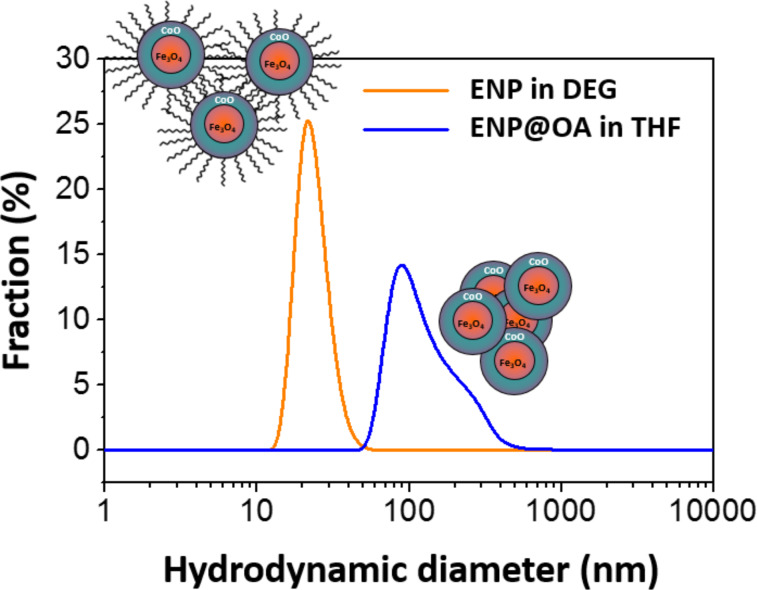
Size distributions of ENPs, in polyol solvent (diethylene glycol) and functionalized by oleic acid, dispersed in tetrahydrofuran solvent (THF), obtained by DLS.

Figure 7 shows the variation of the size distribution of the magnetic particles as a function of the surface state (with or without oleic acid), determined by dynamic light scattering (DLS) measurements. The distribution is found to be quite polydisperse when ENPs are purified by centrifugation from the polyol and narrower when they are coated with OA. Moreover, the average diameter is found to be ≈100 nm for the former and 25 nm for the latter, which shows that the functionalization by OA through ligand exchange leads to a better separation of exchange-biased magnetic particles. Then, PMMA chains were grown from the surface of ENP-Br by the experimental procedure described previously [15].

**Figure 8:**
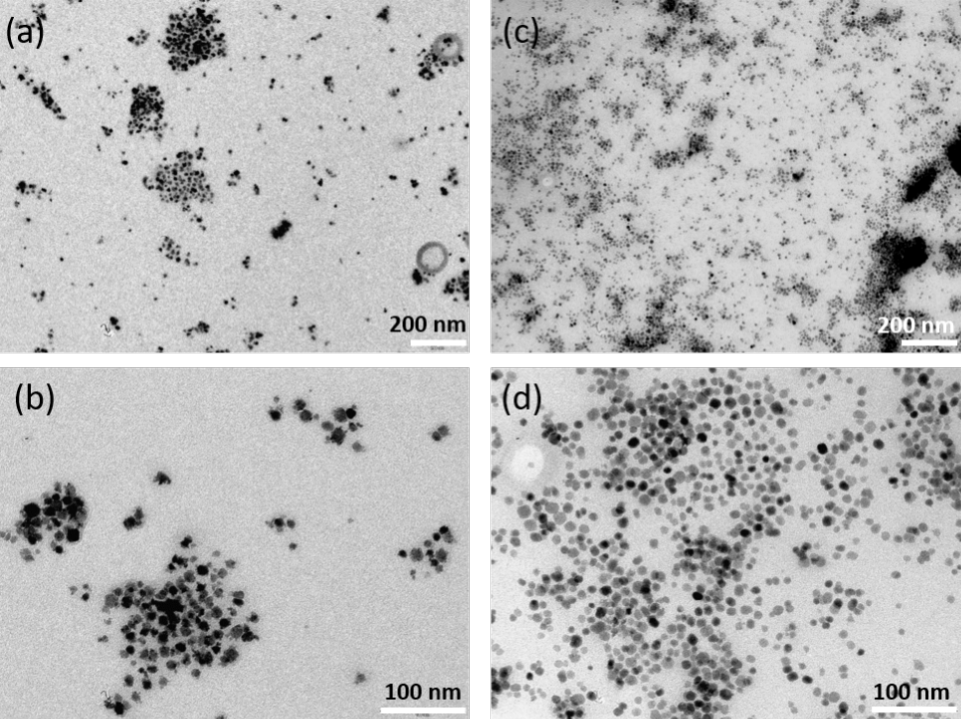
TEM images of magnetic ENPs decorated with PMMA brushes: (a,b) ENP-Br obtained through direct grafting of ATRP initiator after 3 h polymerization; (c,d) ENP-Br obtained by ligand exchange (oleic acid/ATRP initiator) under the same conditions.

Figure 8 presents the TEM images. Better-separated ENPs are recovered when oleic acid is used, and they are, consequently, more adapted for applications in flexible polymer devices.

### Assembly of PMMA-decorated magnetic nanoparticles

**Figure 9:**
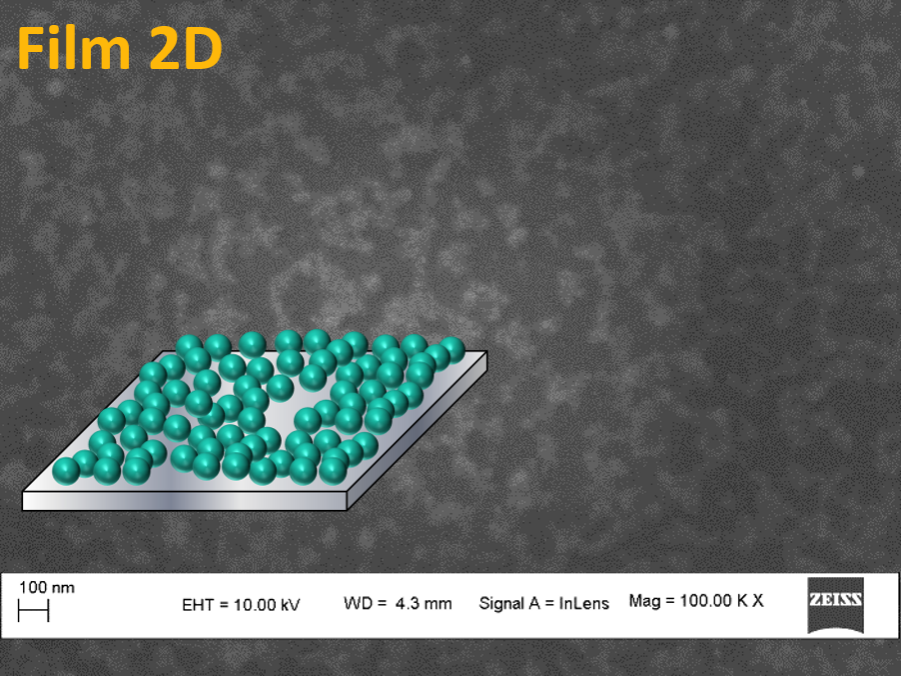
Thin films prepared by drop casting of a suspension of ENP-PMMA in THF.

Finally, we assembled PMMA-functionalized ENPs prepared by the ligand exchange procedure and dispersed in THF. Thin films were prepared by drop casting on silicon wafers and SEM images were recorded. Interestingly, Figure 9 depicts an incomplete monolayer of ENPs (with a scheme presented in the insert).

**Figure 10:**
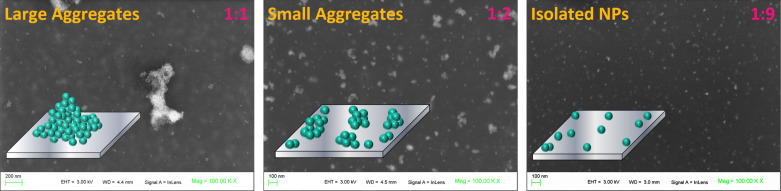
SEM images of various assemblies of ENP-PMMA, obtained by drop casting from THF suspension with ENP/PMMA ratios of 1:1, 1:2 and 1:9.

Then, ENP-PMMA was introduced in a solution of PMMA in THF, where the ENP/PMMA ratio was varied from 1:1 to 1:9. Large aggregates were obtained at higher concentrations of ENPs and small aggregates or isolated ENPs at higher dilutions. SEM images (Figure 10) present well-dispersed ENPs in both cases, suggesting that the ENP-PMMA NPs are very well separated and can be dispersed and assembled in a controlled manner, thanks to the polymer grafted on the ENP surface.

The production of such nanoparticles thus opens the way to various applications such as: (i) the fundamental study of the magnetic properties of the various assemblies of these magnetic ENPs. There is still intensive research to be done on the control of the magnetic properties of magnetic films, either comprised of nanocomposites or not [37], via the spatial ENP orientation [28,38] to be combined with polymer processing [39–40].

**Figure 11:**
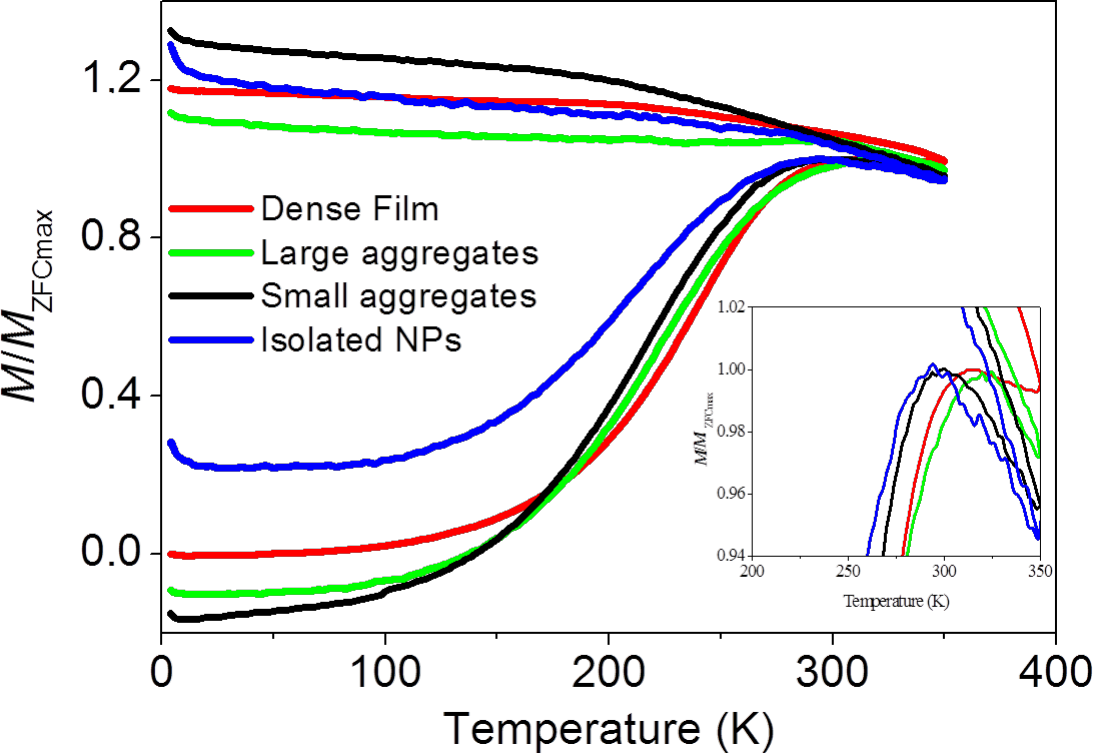
Thermal variation of the normalized dc magnetic magnetization measured in zero-field cooling (ZFC) conditions for the assembled PMMA-decorated ENP sample series (see Figure 10). Details around the maximum of the magnetization is given in the inset.

As a preliminary result, focusing on these PMMA-decorated ENP assemblies, a net decrease of the blocking temperature value, defined as the critical temperature at the relaxed/blocked magnetic states transition, was observed when the ENP dilution ratio was increased (Figure 11). Such behaviour is quite common for superparamagnetic single-domain nanoparticles, and it is here respected in the case of exchange-biased nanoparticles.

It is clear that the material processing approach we proposed here, namely, the controlled surface polymerization of oxide-based ENPs, is able to tune their magnetic properties. Further magnetic measurements are still in progress to fully appreciate the dipolar interaction effect on the exchange bias and the influence of NP assembly on the exchange field.

## Conclusion

We described the functionalization of 10 nm exchange-biased Co*_x_*Fe_3−_*_x_*O_4_@CoO core@shell NPs by two different polymers, poly(methyl methacrylate) (PMMA) and polystyrene (PS), using radical-controlled polymerization under various processing conditions. We evidenced through TGA, IR spectroscopy and XPS measurements that polymer chains were efficiently grafted onto the nanoparticles using either the direct grafting or the “all in solution” process. In the TEM images we have also observed that very little aggregation occurs. Nevertheless, the alternative two-step strategy leads to better dispersed polymer-decorated magnetic particles, and the resulting nanohybrids can be considered as valuable building blocks for flexible, magnetic polymer-based devices. We also developed various assemblies by varying the dilution of the ENP suspension in THF, where the assemblies varied from small aggregates to isolated ENPs at the surface of silicon substrates. These ENPs allow for the preparation of flexible, functional, hybrid PMMA-ENP films with enhanced properties (e.g., magnetic, mechanical).

## Experimental

### Hybrid synthesis

PMMA chain growth by ATRP (direct ligand exchange for ATRP initiator grafting) was used as described in [15]: ENPs and 2-phosphonooxy-2-bromo-2-methylpropanoate were introduced in THF and sonicated at room temperature (RT). The resulting nanohybrids were then dispersed in acetonitrile before adding *N*,*N*,*N*′,*N*′,*N*′′-pentamethyldiethylenetriamine (PMDTA) methyl methacrylate. The mixture was degassed with argon and mechanically stirred for 1 h. CuBr was added and the solution was heated around 30 °C and sonicated under inert atmosphere. The polymerization time was tuned from 1 to 3 h to influence the length of the polymer chains. The reaction was quenched by opening the system and diluting the solution with THF and hexane. Hairy, hybrid ENPs were recovered by centrifugation and washing with THF. They were referred to as ENP-PMMA-h, where h corresponds to the polymerization time in hours.

PS chain growth (direct ligand exchange for ATRP initiator grafting) proceeded by first dispersing 80 mg of ENP-Br in 37 mL of toluene before adding 8.2 μL of PMDTA, 4.8 mL of styrene and 11.4 mL of malonitrile in order to arrive at the ratio styene/initiator/malonitrile/PMDETA/CuBr 1000:1:4:1:1. The mixture was degassed with argon and mechanically stirred for at least 1 hour. 5.7 mg of CuBr were added and the solution was heated at 90 °C and mechanically stirred under argon. The polymerization time was varied from 18 to 24 h. Finally, the reaction was quenched by opening the system to the atmosphere. The resulting hybrids were referred to as ENP-PS-h, where h corresponds to the polymerization time in hours.

PMMA chain growth (two-step ATRP initiator grafting, ligand exchange by phase transfer) was accomplished using 150 mg of ENPs, dispersed in 45 mL of diethylene glycol, which was added to 75 mL of a solution of oleic acid in toluene (10% v/v) and sonicated for 15 min and left overnight. The OA-functionalized ENPs were recovered from the toluene layer and 375 mg of 2-phosphonooxy-2-bromo-2-methylpropanoate were added to the suspension and sonicated for 24 h. Toluene was then removed and the functionalized ENPs were recovered after several washings with THF.

### Hybrid characterization

The different reaction steps were monitored by ATR-FTIR on a Thermo Nicolet 8700 spectrometer equipped with a diamond crystal (50 scans, 4 cm^−1^ resolution). Thermogravimetric analyses (TGA) were performed in air on a Labsys-Evo device with a heating rate of 10 °C min^−1^. X-ray photoelectron spectroscopy (XPS) measurements were performed on as-prepared and functionalized ENPs using a Thermo VG ESCALAB 250 instrument equipped with a micro-focused, monochromatic Al Kα X-ray source (1486.6 eV) and a magnetic lens. The X-ray spot size was 500 µm (15 kV, 150 W). The spectra were acquired in the constant analyser energy mode with pass energies of 150 and 40 eV for the general survey and the narrow scans, respectively. The samples were fixed on sample holders and out-gassed in the fast entry airlock (2 × 10^−7^ mbar). The Avantage software package was used for data acquisition and processing. The C 1s line of 285 eV was used as the reference to correct the binding energies. Scanning electron microscopy (SEM) and transmission electron microscopy (TEM) were performed on Supra40 ZEISS FEG-SEM and JEOL-100-CX II TEM microscopes, operating at 5.0 and 100 kV, respectively.
